# Identification of key biomarkers of telomere-related genes in diabetic nephropathy via bioinformatic analysis

**DOI:** 10.3389/fgene.2026.1566012

**Published:** 2026-02-04

**Authors:** Dan Yu, Zhipeng Feng, Huan Yao, Ke An

**Affiliations:** Department of Nephrology, Affiliated Hospital of Guizhou Medical University, Guiyang, China

**Keywords:** biomarker, diabetic nephropathy, machine learning, single-cell analysis, telomere-related genes

## Abstract

**Background:**

Diabetic nephropathy (DN) is a major cause of end-stage renal disease. Understanding the molecular mechanisms underlying DN is crucial for developing new therapeutic targets and diagnostic biomarkers.

**Methods:**

We utilized microarray data from the GEO database to identify differentially expressed genes related to DN. Machine learning algorithms, including LASSO regression and SVM-RFE, were employed to screen and validate telomere-related genes. We also predicted the transcription factors of the significant genes. Subsequently, correlation analysis and Receiver Operating Characteristic diagnostics were performed on the key genes, along with validation using external datasets. Additionally, GSEA enrichment analysis and immune infiltration analysis were conducted. Furthermore, we analyzed the expression of significant genes in cell subgroups using single-cell sequencing technology. Finally, key genes were validated in DN kidney biopsy tissues and normal kidney biopsy tissues.

**Results:**

Through differential analysis and machine learning screening, we identified a total of 14 differentially expressed genes related to telomeres, among which TRIM22, ELOVL4, NLGN4X, and FOSB were highlighted as key genes. We also predicted seven related transcription factors (BCLAF1, HNRNPL, TAF15, STAT1, SRSF9, SAFB2, PTEN). The key gene TRIM22 showed a high correlation with NLGN4X, ELOVL4, and NLGN4X. ROC diagnostics demonstrated sufficient diagnostic accuracy in both the test and validation sets. GSEA enrichment analysis and immune infiltration analysis revealed significant differences among immune cells, such as PC cells, and preliminary expression validation was conducted using single-cell analysis (for example, TRIM22 exhibited high expression levels in EDC, PEC, MES, and IMC). Finally, we performed RT-PCR between DN samples and control samples, finding that the expression levels of key genes in both groups were consistent with the trends predicted by bioinformatics, indicating that these genes may serve as potential diagnostic biomarkers and therapeutic targets.

**Conclusion:**

This study provides a comprehensive analysis of telomere-related DEGs in DN, enhancing our understanding of DN pathogenesis. The identified key genes offer potential for new diagnostic and therapeutic strategies, warranting further investigation into their biological roles in DN.

## Graphical Abstract

**Figure:**
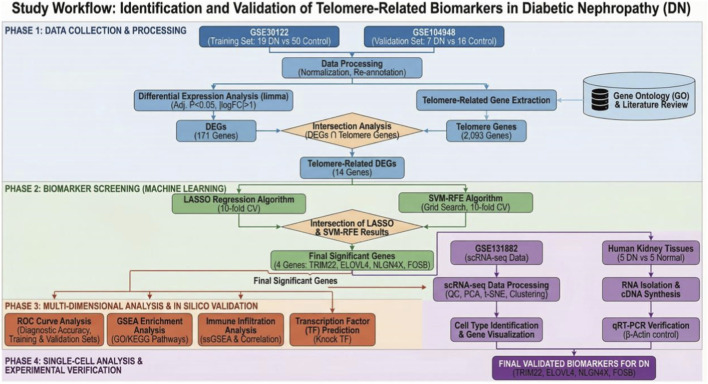

## Introduction

According to the International Diabetes Federation (IDF), about 463 million people worldwide were living with diabetes in 2019, which is forecasted to rise to 578 million by 2030 and 700 million by 2045 (Williams et al., 2020). With the increasing prevalence of diabetes worldwide, diabetic nephropathy (DN) has become the leading cause of end-stage renal disease, causing approximately 30%–50% of ESRD (Ruiz-Ortega et al., 2020). Patients with diabetes and DN face significantly higher risks of cardiovascular events, disability, and mortality, imposing substantial economic burdens on families and society (Dugbartey, 2017). The incidence of diabetic nephropathy is still rising globally and has become a major challenge in the field of public health. Therefore, studying the pathogenesis and therapeutic targets related to diabetic nephropathy is of great significance. Studying its pathogenesis helps to deeply understand how diabetes damages the structure and function of the kidneys through various pathways (such as hyperglycemia, oxidative stress, inflammatory response, apoptosis, etc.). Currently, the treatment of diabetic nephropathy mainly focuses on blood glucose control and traditional anti-inflammatory drugs, but the efficacy of these methods is limited. By studying the pathogenesis, new therapeutic targets can be discovered, thus developing more effective treatment strategies. Meanwhile, it helps to identify different subtypes and key biomarkers of the disease, thus achieving precision medicine. In addition, it can also delay the progression of the disease, reduce the incidence of end-stage renal disease, and thus significantly improve the prognosis of patients.

Telomeres, composed of repetitive TTAGGG DNA sequences and shelterin complexes, are crucial for maintaining chromosome stability by protecting chromosome ends (Shay and Wright, 2019). These structures shorten with each cell division and in certain disease states. Telomere shortening or dysfunction can lead cells to enter a senescent state (Guo et al., 2020). In diabetic nephropathy, the hyperglycemic environment accelerates telomere shortening, leading to premature senescence of renal cells (such as mesangial cells and podocytes) (Guo et al., 2020). Aged renal cells secrete a variety of inflammatory factors and extracellular matrix components, promoting renal tissue fibrosis and inflammatory responses, which are important pathological features of diabetic nephropathy. In diabetic nephropathy, hyperglycemia itself can induce oxidative stress, leading to an increase in the generation of reactive oxygen species (ROS). Telomere-associated dysregulation further exacerbates this state of oxidative stress, damaging the mitochondria within cells and causing changes in mitochondrial morphology and function, such as increased mitochondrial fission and reduced fusion. This abnormality in mitochondrial dynamics can lead to mitochondrial respiratory chain dysfunction, further increasing the generation of ROS and forming a vicious cycle (Han et al., 2018). This continuous oxidative stress can damage the DNA, proteins and lipids of renal cells, leading to cell dysfunction and apoptosis, thereby aggravating the pathological process of diabetic nephropathy. Moreover, mitochondrial dysfunction can also affect the energy metabolism of cells, resulting in insufficient energy supply to cells and affecting the normal function of renal cells. Secondly, telomere-associated dysregulation can promote the apoptosis of renal cells by activating apoptosis-related proteins such as p53 (Ma et al., 2020). The increase in this type of apoptosis can lead to renal tissue damage and dysfunction, accelerating the progression of diabetic nephropathy. In addition, telomere shortening can induce cells to secrete a variety of inflammatory factors (Wang et al., 2025), such as interleukin-6 (IL-6), tumor necrosis factor-α (TNF-α), etc. These inflammatory factors will further activate the inflammatory response in renal tissue, resulting in mesangial cell proliferation, extracellular matrix accumulation and glomerulosclerosis. The inflammatory response promotes the generation of oxidative stress, and oxidative stress also activates the apoptotic signaling pathway within cells, further aggravating apoptosis and forming a vicious cycle. In conclusion, telomere-associated dysregulation accelerates and exacerbates the pathophysiological process of diabetic nephropathy by accelerating cellular senescence, intensifying oxidative stress, promoting apoptosis, activating inflammatory responses and affecting mitochondrial function.

Recent advances in genomics and bioinformatics have enabled researchers to gain a deeper understanding of the mechanisms by which telomeres operate in DN. However, there is currently a lack of research on the significance of telomeres in DN. Therefore, the aim of this study is to use single-cell RNA sequencing combined with bulk sequencing technology to identify telomere-related biomarkers in DN. This approach has the potential to delve into the underlying mechanisms of DN and aid in the discovery of potential therapeutic targets for future interventions.

## Methods

### Microarray data

The GEO database (http://www.ncbi.nlm.nih.gov/geo/) served as a public repository for functional genomics data, encompassing microarray, next-generation sequencing, and various high-throughput functional genomics datasets. We downloaded the whole blood transcriptome profile dataset GSE30122 from GEO. After standardization and re-annotation, the probes were converted into their corresponding gene symbols. The training set, GSE30122, comprised 19 samples with diabetic nephropathy (DN) and 50 control samples. The validation set, GSE104948, included 7 DN samples and 18 control samples.

### Identification of telomere-related DEGs

We utilized the ‘limma’ package in R software to screen for differentially expressed genes (DEGs) between DN and non-DN samples, identifying telomere-related DEGs. We considered an adjusted P-value <0.05 and an absolute log fold change (logFC) > 1 as statistically significant. A total of 2,093 telomere-related genes were extracted from the Gene Ontology Database (http://geneontology.org/), specifically selecting genes and gene products annotated with terms directly related to telomeres and telomere maintenance. When screening telomere-associated genes, we primarily relied on entries directly related to telomeres in the Gene Ontology (GO) database, including but not limited to GO:0000723 (telomere repair), GO:0003691 (telomere binding), GO:0007004 (telomere maintenance), GO:0032200 (telomere organization), and GO:0010833 (telomere elongation). We included all genes annotated with the above terms in the initial candidate pool. Additionally, we consulted recent high-impact review articles and included genes confirmed by experimental evidence in their Supplementary Data or Methods sections to be associated with telomere regulation, the telomerase complex, telomeric proteins, and related pathways. We merged the genes from all GO entries mentioned above and supplemented them with telomere-associated genes reported in the literature, which resulted in a final set of 2,093 genes (S1). The genes that overlapped between the two datasets were classified as telomere-related DEGs.

### Immune infiltration analysis

We calculated the proportions of infiltrating immune cell subsets using the ‘ssGSEA’ package. To ensure the accuracy and reproducibility of the ssGSEA analysis based on “mmc3”, we performed normalization of the bulk RNA-seq data prior to the analysis. Given the extensive computation of correlations between multiple key genes and numerous immune cell subpopulations, the risk of false positives due to multiple testing is significantly elevated. Therefore, all p-values from our correlation analyses have undergone Benjamini–Hochberg correction for False Discovery Rate (FDR), and the results are presented using the corrected q-values.

### Machine learning screening and validation of gene biomarkers

We employed two machine learning methods: the LASSO regression algorithm and the SVM-RFE algorithm, to identify genes highly associated with DN. The LASSO regression algorithm, implemented using the ‘glmnet’ package, was used for initial feature selection. During the LASSO analysis, optimal model complexity was determined by selecting the best λ value through 10-fold cross-validation. A larger λ value signifies stronger regularization, leading to the selection of fewer independent variables. For the Support Vector Machines Recursive Feature Elimination (SVM-RFE) algorithm, we applied a strategy involving SVM and Recursive Feature Elimination (RFE) for refining gene selection. This process iteratively identified important genes. To optimize the core parameters of the SVM-RFE, a grid search approach, coupled with 10-fold cross-validation, was performed. This systematic evaluation explored various configurations related to the number of features to eliminate in each iteration (e.g., 5, 10, 15, 20, 25, 30) and the halve. above threshold (e.g., 10, 20, 30, 40, 50, 60, 70, 80, 90, 100). The parameter combination that demonstrated superior performance in terms of classification accuracy and stability was identified as removing 10 genes per iteration with a halve. above threshold of 30. Subsequently, a feature selection evaluation was conducted using a nested approach. Different feature combinations were assessed for their predictive performance, and prediction errors were computed to quantify the effectiveness of the selected features.

Finally, the genes identified by both the LASSO and SVM-RFE methods were intersected to derive the final set of significant genes. Receiver Operating Characteristic (ROC) curves were constructed to assess their predictive power. Additionally, transcription factor (TF) prediction for the identified significant genes was performed using the Knock TF-Analysis tool.

### GSEA enrichment analysis for key genes

To explore the biological pathways and functions related to key genes, we performed gene set enrichment analysis (GSEA) using R packages including cluster Profiler (version 4.0), Reactome PA, org. Hs. e.g.,.db, enrichplot, and msigdbr. The analysis workflow was as follows: First, we divided samples into high and low expression groups based on the median expression level of target genes. Then, we calculated the log2 mean difference between the high and low expression groups, which served as the ranking metric for subsequent GSEA. To ensure ratio stability, we applied a minimal value correction to very low expression values. We ranked all genes by logFC to obtain a ranked gene list and converted gene symbols to ENTREZID format. For the GO analysis, we selected three major categories: “Biological Process (BP),” “Cellular Component (CC),” and “Molecular Function (MF).” For KEGG analysis, we used the human KEGG database (“hsa”). The Reactome analysis employed the Reactome human pathway database. During the analysis, we set minimum and maximum gene set sizes to ensure the rationality of pathway enrichment. A uniform significance threshold of FDR-corrected p-value <0.05 was employed. Finally, we visualized the main enriched pathways using the “dotplot” function from the enrichplot package.

### Identification of renal cells

We downloaded single-cell sequencing data from three diabetic nephropathy samples in GEO database GSE 131882, which included 9,270 cells and 26,052 genes. Initially, we filtered the data by setting the parameters min. cells = 5, min. features = 300, 200 < nFeature_RNA <2500, and percent. mt < 5. Subsequently, we normalized the data using the NormalizeData function. Normalization specifically involved dividing each cell’s gene expression counts by the total counts and then multiplying by 10,000, allowing the normalized expression values for each cell to be compared on a similar scale. This ensures that the expression levels between different cells more accurately reflect the actual biological differences. We then used the FindVariableFeatures function to identify the top 1,500 genes with the highest standardized variance. Next, we performed data scaling and principal component analysis (PCA) using ScaleData and RunPCA, setting npcs = 20. Afterward, we selected the optimal principal component (PC) values based on the results from ElbowPlot and JackStraw, and sequentially obtained clustering and t-SNE values using FindNeighbors, FindClusters, and RunTSNE. Finally, we annotated the clusters based on marker genes reported in the literature and visualized the target genes using t-SNE.

### RNA isolation and quantitative real-time polymerase chain reaction (qRT-PCR)

We collected kidney tissue from patients with diabetic nephropathy (n = 5) and minimal change nephropathy as a control group (n = 5) from the Affiliated Hospital of Guizhou Medical University. All participants have signed the informed consent form and have obtained the consent of the hospital ethics committee. All renal biopsy tissue samples were lysed using TRIzol reagent (Sichuan Science and Technology Biological Co., Ltd.) to extract total RNA according to the manufacturer’s instructions. The concentration and purity of the RNA solution were quantified using a K2800 nucleic acid analyzer (Beijing Kaio Technology Development Co., Ltd.). The extracted RNA was reverse transcribed into cDNA using the SureScript First-Strand cDNA Synthesis Kit (Genecopoeia, Guangzhou, China), followed by qRT-PCR reactions. The qRT-PCR reaction system included 3 µL of reverse transcription product, 5 µL of 5× All-In-One MasterMix (with AccuRT Genomic DNA Removal kit, abm), and 1 µL of forward primer and 1 µL of reverse primer. The PCR reactions were performed in an automated medical PCR analysis system (Shanghai Hongshi Medical Technology Co., Ltd. SLAN-96S) under the following conditions: initial denaturation at 95 °C for 1 min, followed by 40 cycles, each consisting of incubation at 95 °C for 20 s, 55 °C for 20 s, and 72 °C for 30 s. The primer information is as follows: the forward primer for β-Actin is AAT​CTG​GCA​CCA​CAC​CTT​CTA​CAA, and the reverse primer is GGA​TAG​CAC​AGC​CTG​GAT​AGC​AA; the forward primer for TRIM22 is AAC​CAC​GGA​GCA​CTC​ATC​TAC​AAG, and the reverse primer is GGC​AGT​TCC​AAG​GAT​TGA​AAT​ACG​G; the forward primer for ELOVL4 is TCC​ACG​GCA​CTC​AAC​GAC​AC, and the reverse primer is CCA​GCC​ACA​CAA​ACA​GGA​GAT​AAA​G; the forward primer for NLGN4X is TGC​TAT​GGC​TTC​CTT​TGT​TGT​TCA​C, and the reverse primer is ACT​GGA​TAC​TGT​GCT​TGG​CTG​TC; the forward primer for FOSB is GCA​ACC​CAC​CCT​CAT​CTC​TTC​C, and the reverse primer is CGC​CAC​TGC​TGT​AGC​CAC​TC. All primers were synthesized by Sichuan Science and Technology Biological Co., Ltd. The GAPDH gene was used as an internal control to determine the relative expression levels of significant genes using the 2^−ΔΔCT^ method. The experiments were repeated three times independently. A paired t-test was used to compare the statistical differences between the DN group and the normal group.

### Statistical analysis

All bioinformatics analyses were conducted using R software. We performed difference analysis and correlation analysis using the Wilcoxon and Pearson tests, respectively. A P-value <0.05 was deemed statistically significant.

## Results

### Transcriptomic profiling reveals telomere dysfunction in DN

**FIGURE 1:**
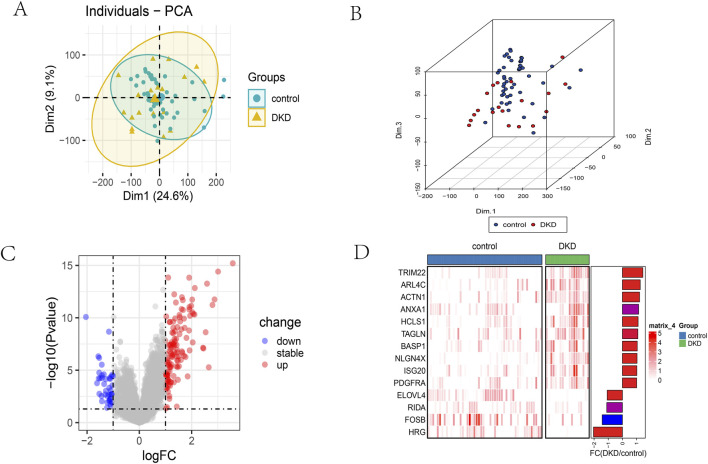
Identification of telomere‐related DEGs in DN. 2D **(A)** and 3D **(B)** PCA of the healthy and DN samples. **(C)** Volcano plot of DEGs between the normal and DN samples. **(D)** Heatmap of DEGs between the normal and DN samples.

Nephropathy Telomere attrition is a hallmark of cellular senescence and has been implicated in the progression of chronic kidney disease. To systematically investigate telomere-related alterations in DN, we first screened the GSE30122 dataset, identifying 171 differentially expressed genes (DEGs) (129 upregulated, 42 downregulated) (Figures 1A–C). Given the complexity of the DN transcriptome, we sought to isolate pathways specifically linked to telomere maintenance. By intersecting these global DEGs with a curated library of 2,093 telomere-associated genes derived from GO annotations and literature, we identified a subset of 14 telomere-related DEGs (Figure 1D). This step significantly narrowed the candidate pool to genes biologically relevant to telomere homeostasis.

### Ensemble machine learning identifies a robust four-gene diagnostic signature

**FIGURE 2:**
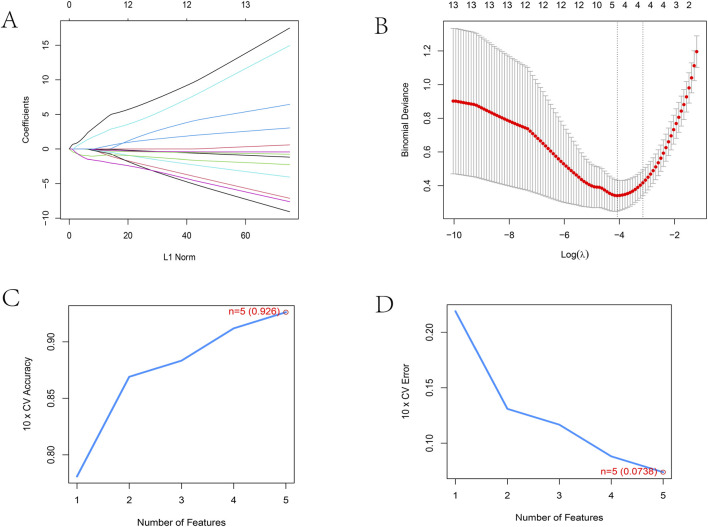
Identification of biomarkers based on machine learning algorithms. **(A)** A LASSO regression analysis of 14 telomere‐related DEGs. **(B)** A cross‐validation procedure is used to optimize the parameter selection in the LASSO regression. **(C,D)** Per‐cell prediction true and error value change curves by SVM‐RFE analysis.

To distill these 14 candidates into a clinically robust diagnostic signature, we employed two independent machine learning algorithms. First, LASSO regression, which excels at regularization to prevent overfitting, identified a subset of 5 genes (FOSB, HRG, TRIM22, ELOVL4, NLGN4X) with optimal λ selection (Figures 2A,B). In parallel, Support Vector Machine-Recursive Feature Elimination (SVM-RFE) was applied to rank features based on classification power, selecting a 5-gene set (TRIM22, ELOVL4, BASP1, NLGN4X, FOSB) (Figures 2C,D). To ensure maximum reliability, we intersected the results from both algorithms, yielding four key consensus genes: TRIM22, ELOVL4, NLGN4X, and FOSB.

**FIGURE 3:**
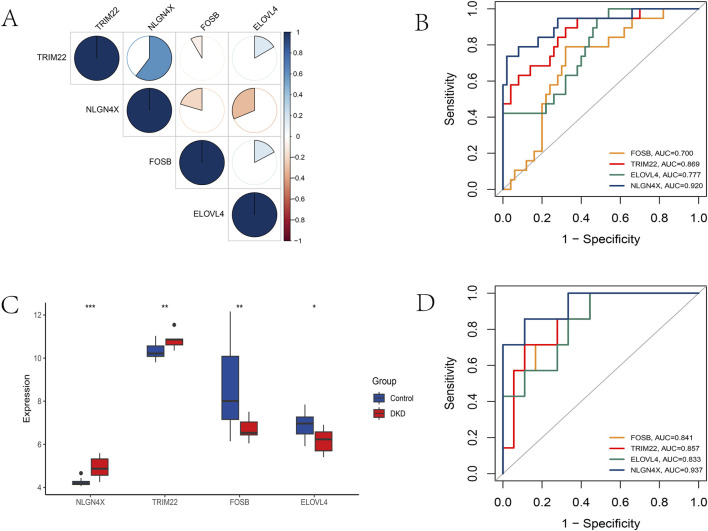
The key genes analysis and validation. **(A)** Correlation analysis between related genes. **(B)** ROC of key genes in the training set. **(C)** Comparison of key gene expression between the two groups in the validation set. **(D)** ROC of key genes in the validation set.

A network analysis of significant genes and their coexpressed genes revealed a high correlation between TRIM22 and NLGN4X, as well as between ELOVL4 and NLGN4X (Figure 3A). We then evaluated the diagnostic performance of this four-gene signature. In the training set, ROC analysis demonstrated high predictive accuracy, with NLGN4X achieving an AUC of 0.920 (Figure 3B). Crucially, we validated these findings in an independent external cohort (GSE104948). Consistent with the training data, NLGN4X and TRIM22 were upregulated, while FOSB and ELOVL4 were downregulated in DN patients (Figure 3C). The validation set ROC curves confirmed their robust diagnostic potential (AUC: NLGN4X = 0.937, TRIM22 = 0.857, FOSB = 0.841, ELOVL4 = 0.833) (Figure 3D). Internal 3-fold cross-validation further supported the stability of this model (Supplementary Figure S2).

### TF analysis

**FIGURE 4:**
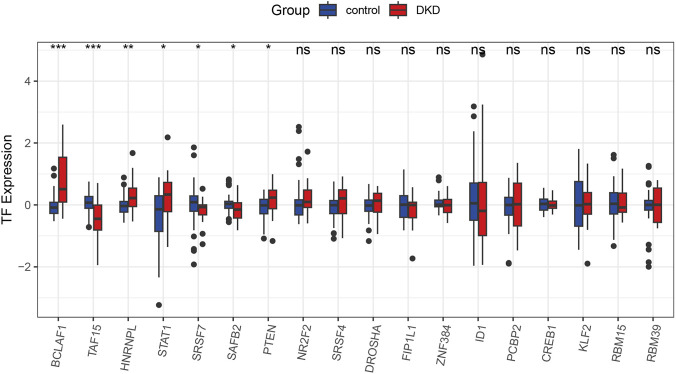
Comparison of the TF expression. Comparison of the TF expression between control and DN samples. *P < 0.05, *P* < 0.01, *P < 0.001.

Based on significant genes, 18 TFs were predicted by Knock TF-Analysis. There were statistically significant differences in BCLAF1, HNRNPL, TAF15, STAT1, SRSF9, SAFB2, PTEN between DN and control groups (Figure 4).

### Immune infiltration analysis

**FIGURE 5:**
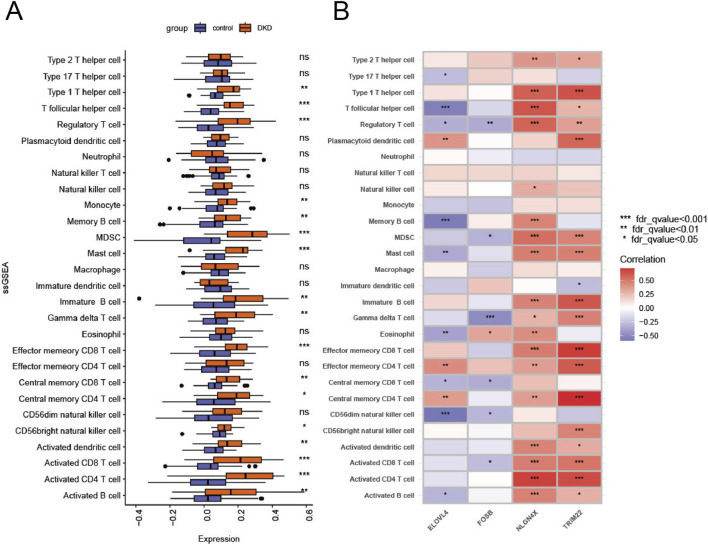
Comparison of the immune activity between control and DN samples. **(A,B)** The immune infiltration of DN samples was analyzed by ssGEEA. *P < 0.05, P < 0.01, *P < 0.001.

Chronic inflammation and immune cell infiltration are central drivers of DN progression. To understand how these telomere-related genes might interact with the immune microenvironment, we utilized ssGSEA to map the immune landscape. We observed widespread perturbation in immune subsets, including increased infiltration of Activated CD4^+^ T cells, Macrophages, and MDSCs in DN patients (Figure 5A). Correlation analysis revealed distinct regulatory patterns: TRIM22 and NLGN4X showed strong negative correlations with Type 2 T helper cells and Immature B cells (Figure 5B). This suggests that the upregulation of TRIM22 and NLGN4X in DN may actively shape a pro-inflammatory and senescent immune milieu, potentially through telomere-mediated stress responses.

### GSEA enrichment analysis

**FIGURE 6:**
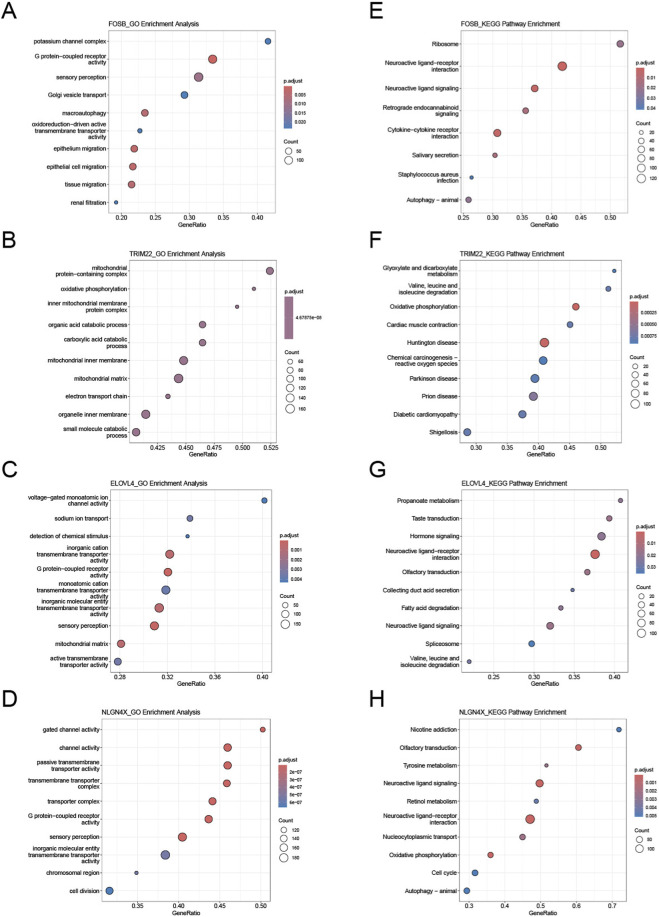
GSEA enrichment analysis. **(A–D)**: Top 10 GO enrichment results from ssGSEA for FOSB, TRIM22, ELOVL4, and NLGN4X, respectively; **(E–H)**: Top 10 KEGG enrichment results from ssGSEA for FOSB, TRIM22, ELOVL4, and NLGN4X, respectively.

The ssGSEA analysis results for the four genes TRIM22, ELOVL4, NLGN4X, and FOSB showed that in terms of GO function enrichment, they were primarily and significantly enriched in mitochondrial protein complex, voltage-gated monovalent inorganic cation channel activity, gated channel activity, and potassium channel complex, respectively (Figures 6A–D). In terms of KEGG pathway enrichment, they were significantly enriched in glyoxylate and dicarboxylate metabolism, propanoate metabolism, nicotine addiction, and ribosome, respectively. These findings suggest that the aforementioned genes may play important roles in biological processes related to energy metabolism, ion channel regulation, and neural activity (Figures 6E–H).

### Identifications of renal cells

**FIGURE 7:**
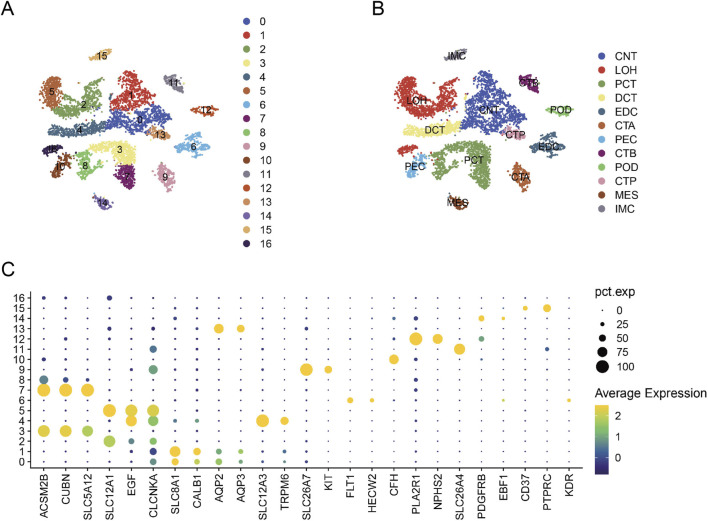
Renal Cell Type Analysis. **(A)** PCA analysis results. **(B)** Identified cell subgroups. **(C)** Distribution of different markers across different clusters (Proximal tubular convoluted cells (PCT), cells of the loop of Henle (LOH), connecting tubule cells (CNT), principal cells of the collecting duct (CTP), distal convoluted duct cells (DCT), intercalated cell A from the collecting duct (CTA), glomerular endothelial cells (EDC), parietal epithelial cells (PEC), podocytes (POD), intercalated cell B from the collecting duct (CTB), mesangial cells (MES), and immune cells (IMC) were manually identified).

After performing data filtering, we obtained a total of 7840 cells. We identified a total of 17 clusters after selecting the optimal PC value of 15 (Figure 7A). We manually identified proximal convoluted tubule cells, Henle’s loop cells, connecting tubule cells, principal cells of the collecting duct, distal convoluted tubule cells, intercalated cells of the collecting duct type A, endothelial cells, epithelial cells of the tubule wall, podocytes, intercalated cells of the collecting duct type B, mesangial cells, and immune cells based on known markers (Figure 7B). We presented the distribution of the markers used in this study across different clusters s (Figure 7C).

**FIGURE 8:**
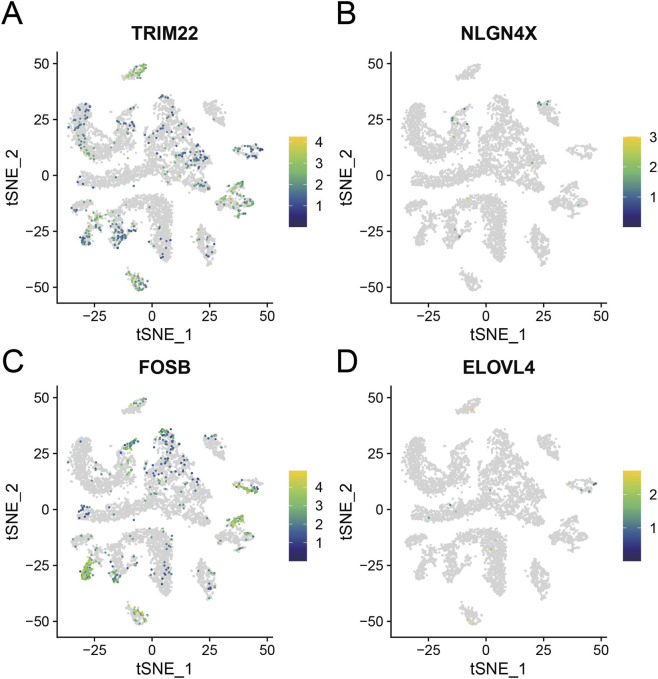
Distribution of TRIM22 **(A)**, NLGN4X **(B)**, FOSB **(C)**, and ELOVL4 **(D)** in different cell subgroups.

We used tSNE to visualize the distribution of TRIM22, NLGN4X, FOSB, and ELOVL4 in different cell subgroups (Figures 8A–D). We observed that TRIM22 exhibited high expression levels in EDC, PEC, MES, and IMC, while FOSB displayed higher expression in CNT, EDC, PCT, PEC, POD, and MES. In contrast, NLGN4X and ELOVL4 showed lower expression levels across all subgroups.

### Experimental verification using RT-PCR

**FIGURE 9:**
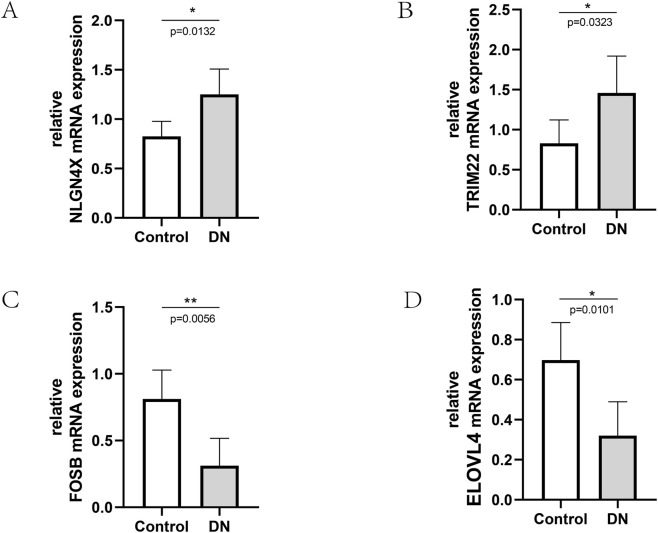
Relative mRNA expression in Control and DN samples. **(A–D)** Relative mRNA expression of NLGN4X, TRIM22, FOSB, and ELOVL4 in Control and DN samples ( $\bar{x}$ ±s, n = 5), *P < 0.05.

We collected kidney tissues from five cases of diabetic nephropathy, using minimal change nephropathy as the control group. Total RNA was extracted from the tissues of each group, and RT-PCR was performed using β-actin as the internal reference. The results, as shown in Figure 9, revealed an increase in NLGN4X and TRIM22 expression and a decrease in FOSB and ELOVL4 expression in diabetic nephropathy kidney tissues compared to the control group (Figure 9). These differences were found to be statistically significant (P < 0.05).

## Discussion

Diabetic nephropathy (DN) has emerged as a prominent contributor to end-stage renal disease globally. Telomeres play a crucial role in the progression of DN, and recent investigations have pinpointed genetic factors involved in its pathogenesis. Nevertheless, the precise molecular mechanisms that drive DN remain elusive. Given the current diagnostic constraints and the lack of effective therapeutic strategies to impede the advancement of DN, there is an urgent necessity to investigate potential early biomarkers for diagnosis and treatment. This study pioneers the examination of telomere-associated genes in the pathogenesis of DN, leveraging microarray databases to uncover genetic variations. Progress in molecular biology has yielded enhanced understanding of the heterogeneity of DN and holds the promise of identifying novel prognostic markers for future therapeutic interventions.

In our investigation, one mRNA microarray dataset was scrutinized, leading to the identification of 171 differentially expressed genes (DEGs) between glomerular tissues from 19 DN cases and 50 control samples, comprising 129 upregulated and 42 downregulated genes. After intersecting with telomere-related genes, 14 telomere-associated DEGs were identified. Subsequently, four telomere signature genes pertinent to DN were determined based on the Random Forest Model (RFM) approach: TRIM22, ELOVL4, NLGN4X, and FOSB. Polymerase chain reaction (PCR) analysis was conducted on kidney tissue affected by diabetic kidney disease for further validation.

TRIM22, as an E3 ubiquitin ligase, participates in innate immune responses and inflammatory reactions, potentially regulating glomerular inflammation and cellular injury in Diabetic Kidney Disease (DKD). Hyperglycemia and oxidative stress can further activate FOSB, upregulating inflammatory factors and promoting the expression of genes related to cell proliferation and fibrosis. Our research found that the expression of TRIM22 is increased in patients with diabetic nephropathy, while the expression of FOSB is decreased, suggesting a potential association between TRIM22 and FOSB. However, the specific signaling pathways involved require further investigation. ELOVL4 is responsible for the synthesis of very long-chain fatty acids and influences lipid metabolism. Dysregulation of ELOVL4 leads to abnormal lipid metabolism, which can exacerbate renal injury. TRIM22 may affect lipid metabolism-related proteins, potentially including ELOVL4 expression, through ubiquitination modification. Meanwhile, FOSB can regulate metabolic stress genes, forming a metabolic-inflammatory cross-talk. Although NLGN4X is primarily involved in neural synaptic function, it may mediate cell adhesion and signal transduction in renal cells, influencing cellular communication in DKD. However, the specific mechanisms of its involvement and associations require further study.

Tripartite motif-containing 22 (TRIM22) is a member of the TRIM family of E3 ubiquitin ligase, which can regulate inflammation, apoptosis, and proliferation of multiple types of cells (Medrano et al., 2016). Recent studies have shown that TRIM22 is significantly upregulated in the renal tissue of patients with diabetic nephropathy and in high-glucose-stimulated HK-2 cells. It is closely associated with tubulointerstitial injury and autophagy dysregulation in diabetic nephropathy, and its expression level correlates with the severity of tubular injury (Wang et al., 2025). TRIM22 may promote the release of inflammatory factors by activating inflammatory signaling pathways, thereby exacerbating kidney inflammation and fibrosis. Further, a previous study showed that TRIM22 could alleviate the self-ubiquitination of TNF receptor-associated factor 6 (TRAF6), which was related to the RING domain of TRIM22 (Qiu et al., 2013). The overexpression of TRAF6 was associated with the development of oxidative stress and inflammation (Hu et al., 2020). In diabetic nephropathy, the high-glucose environment can induce the upregulation of TRAF6 expression. TRAF6 promotes the secretion of inflammatory cytokines (such as TNF-α, IL-1β, IL-6) by activating the NF-κB and MAPK signaling pathways, thereby exacerbating renal inflammation. The activation of TRAF6 not only promotes the inflammatory response but also induces the epithelial-mesenchymal transition (EMT) of renal tubular epithelial cells, facilitating the occurrence of renal interstitial fibrosis. This process is one of the important pathological mechanisms in the progression of diabetic nephropathy. The activation of TRAF6 can further induce oxidative stress, damage glomerular mesangial cells and renal tubular epithelial cells, leading to extracellular matrix deposition and renal fibrosis (Yi et al., 2022). In addition, TRAF6 can activate the MAPK pathway (Xiao et al., 2020). Activation of p38-MAPK could also induce DNA double-strand damage (Bomfim et al., 2019; Lian et al., 2014). These results suggested that TRIM22 activated the p38/MAPK pathway by promoting the expression of TRAF6 in these cells. The promotion of the expression of TRAF6 may be due to the mitigatory ubiquitination of TRAF6, which needs to be further explored. All these publications support the analysis results that increased TRIM22 expression in DN patients (Wei et al., 2022). TRIM22 is involved in the inflammatory response and regulates glomerular inflammation and cellular injury in DKD, while hyperglycemia and oxidative stress can further activate FOSB, upregulating inflammatory factors, thereby forming an interaction.

FOSB is a member of the multi-gene Fos family, which regulates cell growth and proliferation. Its homologs include c-Fos, Fra-1, and Fra-2. The members of the Fos family can form activator protein-1 (AP-1) with Jun protein dimer (Prucca et al., 2020). Previous studies have shown that FOS/FOSB activates the expression of IL-6, IL-8, ICAM-1, and MCP-1 in response to hypoxia, which increases cellular inflammatory damage (Wang et al., 2013; Rochette et al., 2013). In the current study, the PPI network showed that FOS/FOSB might be the most important transcriptional factor associated with JMJD1A expression in the combined stimulation of high glucose and hypoxia. RNA sequencing demonstrated that FOS and FOSB have a higher expression in the HUVECs exposed to the combined stimulation of high glucose and hypoxia (Zhao et al., 2021). Previous studies on FOSB have mostly focused on its increased expression related to tumors, inflammation, etc (Liu et al., 2022; Qi et al., 2022). However, our analysis of the GEO database for the DN groups revealed reduced FOSB gene expression. The research by Lin J et al., is consistent with our findings. Their study shows that the expression level of FOS decreases in patients with diabetic nephropathy and diabetic mice. FOSB may participate in the regulation of the inflammatory signaling pathway through the NF-κB pathway, thereby affecting the release of inflammatory factors and thus inhibiting the polarization of M1 macrophages. FOSB may also affect cell proliferation and survival by regulating the MAPK signaling pathway (Lin et al., 2023). FOSB can regulate metabolic stress genes, while ELOVL4 is responsible for the synthesis of very long-chain fatty acids and influences lipid metabolism. Dysregulation of ELOVL4 leads to abnormal lipid metabolism, which can further exacerbate renal injury.

Elongation of Very Long chain fatty acids-4 (ELOVL4) protein, a member of the ELOVL family, is responsible for the biosynthesis of very long chain saturated (VLC-SFA) and polyunsaturated fatty acids (VLC-PUFA). It plays a key role in maintaining vital cellular processes in the body (Yeboah et al., 2021). Different mutations can affect the different biosynthesis of fatty acids, leading to different diseases (Agbaga, 2016). It has been shown that the degradation of VLC-PUFA leads to a decrease in the anti-inflammatory effect and the accumulation of lipid peroxide products in degraded PUFA. Both of the above are procoagulant to inflammatory cells (macrophages) and have direct toxic effects on capillary or retinal tissue, associated with a reduction in ELOVL4 (Tikhonenko et al., 2010). Previous studies on ELOVL4 have mainly focused on the nervous system and retinal lesions. ELOVL4 is an important regulator of synaptic signal transduction and neuronal survival in the central nervous system (CNS). Different mutation groups cause three different neurological diseases in humans (Deák et al., 2019). Conditionally knocked-out ELOVL4 mice exhibit a significant reduction in retinal glycerophospholipids containing very long-chain polyunsaturated fatty acids (VLC-PUFAs), with abnormal accumulations of lipid droplets and lipofuscin-like granules (Harkewicz et al., 2012). All the above studies indicate that ELOVL4 is involved in the lipid metabolism process. Recent studies have found that ELOVL4 may lead to lipid accumulation and metabolic disorders by affecting the lipid metabolism in podocytes. Subsequently, it can cause podocyte dysfunction and cytoskeletal rearrangement, and ultimately result in podocyte injury and death. Podocyte injury can damage the integrity of the glomerular filtration barrier, increase the production of proteinuria, and thus promote the progression of diabetic nephropathy (Fu et al., 2020). ELOVL4 is a key rate-limiting enzyme in the synthesis process of long-chain polyunsaturated fatty acids (VLC-PUFAs), primarily functioning by regulating the anabolic metabolism of fatty acids. Upon ELOVL4 deficiency, both the arachidonic acid pathway and bile acid synthesis pathway exhibit significant downregulation, thereby impacting the production of inflammatory mediators and lipid signaling molecules. The specific mechanisms underlying its role in human physiology remain to be further investigated (Nwagbo et al., 2024).

Neuroligins (NLGNs) are postsynaptic cell adhesion molecules that have crucial roles in synapse function and maturation (Jeong et al., 2017). Expression of NLGN4X increases excitatory synaptic transmission in cultured rat hippocampal neurons (Nguyen et al., 2020). Recent work has indicated that neuroligins are abundantly expressed in blood vessels and implicated in the growth of glioma cells (Samarelli et al., 2014; Venkatesh et al., 2015). NLGN4X is also expressed abundantly in breast cancer tissues. Knockdown of NLGN4X decreased cell proliferation and migration significantly in MDA-MB-231 breast cancer cells, and the depletion of NLGN4X increased cells in both the early and late stages of apoptosis. These studies suggest that NLGN4X is essential for cancer cell survival (Henderson et al., 2017). At present, its specific role in the pathogenesis of diabetic nephropathy remains unclear. NLGNs are mainly involved in synaptic formation and neuronal signal transduction in the nervous system. Recent studies have demonstrated that, they characterize a conserved phosphorylation site, serine 712, on NLGN4X and 4Y. Despite serine 712 being located in a highly conserved region between NLGN4X and 4Y, we observed kinase specificity. PKA exclusively phosphorylates NLGN4X S712, whereas Cdk5 phosphorylates S712 on both NLGN4X and 4Y. NLGN4X S712 phosphorylation regulated spine density, with phosphorylation reducing mature mushroom spines and unphosphorylated S712 increasing spines and enhancing miniature excitatory postsynaptic current frequency (Lehr et al., 2025). Meanwhile, Analysis of the human renal transcriptome and plasma proteome identified NLGN4X as a biomarker for impaired adaptation of the proximal tubule to injury (Wen et al., 2023). In the kidneys, they may affect the progression of kidney diseases by regulating intercellular signaling pathways, influencing the synthesis and degradation of the extracellular matrix, oxidative stress, etc. Similar to the study by Henderson et al. (2017).

It must be admitted that our study has certain limitations. Firstly, this study only utilized retrospective data from the GEO database, and prospective research is needed to confirm these findings. While we validated our results with clinical samples, more experiments are needed for further verification. Future research is expected to include more clinical samples and validate our findings through laboratory analyses. In conclusion, our study identified telomere-related biomarkers in DN, providing a theoretical basis for deeper understanding of DN pathogenesis, exploration of new drug targets, and clinical treatment guidance.

## Conclusion

At present, the non-invasive diagnosis of diabetic nephropathy mainly relies on the urine albumin-to-creatinine ratio (ACR) and estimated glomerular filtration rate (eGFR). However, these indicators often lack sensitivity in the early stage of the disease, making it difficult to detect minor changes in renal function in a timely manner. This study aimed to identify telomere-related DEGs potentially implicated in DN pathogenesis. We identified 14 DEGs and 4 significant genes, suggesting their utility as diagnostic biomarkers and therapeutic targets for DN. Utilizing bioinformatics and machine learning tools, we analyzed gene expression differences between DN patients and healthy controls. Furthermore, TRIM22, ELOVL4, NLGN4X, and FOSB were identified as potential immune-related biomarkers for DN, shedding light on the interplay between immune-related genes and immune cells. These findings enhance our understanding of the molecular mechanisms driving DN progression. Future studies should focus on elucidating the biological roles of these genes in DN. The above biomarkers are expected to provide new directions and targets for the diagnosis and treatment of diabetic nephropathy.

## Data Availability

The datasets presented in this study can be found in the Gene Expression Omnibus repository with the accession numbers GSE30122, GSE104948, and GSE131882.
