# Supplementary material for: Identification of key biomarkers of telomere-related genes in diabetic nephropathy via bioinformatic analysis
Source: Front Genet. 2026 Feb 4;17:1566012. doi: 10.3389/fgene.2026.1566012 (PMC12912712; doi:10.3389/fgene.2026.1566012)
Supplement: Supplementary file 5 [file Presentation2.pptx]

## Slide 1
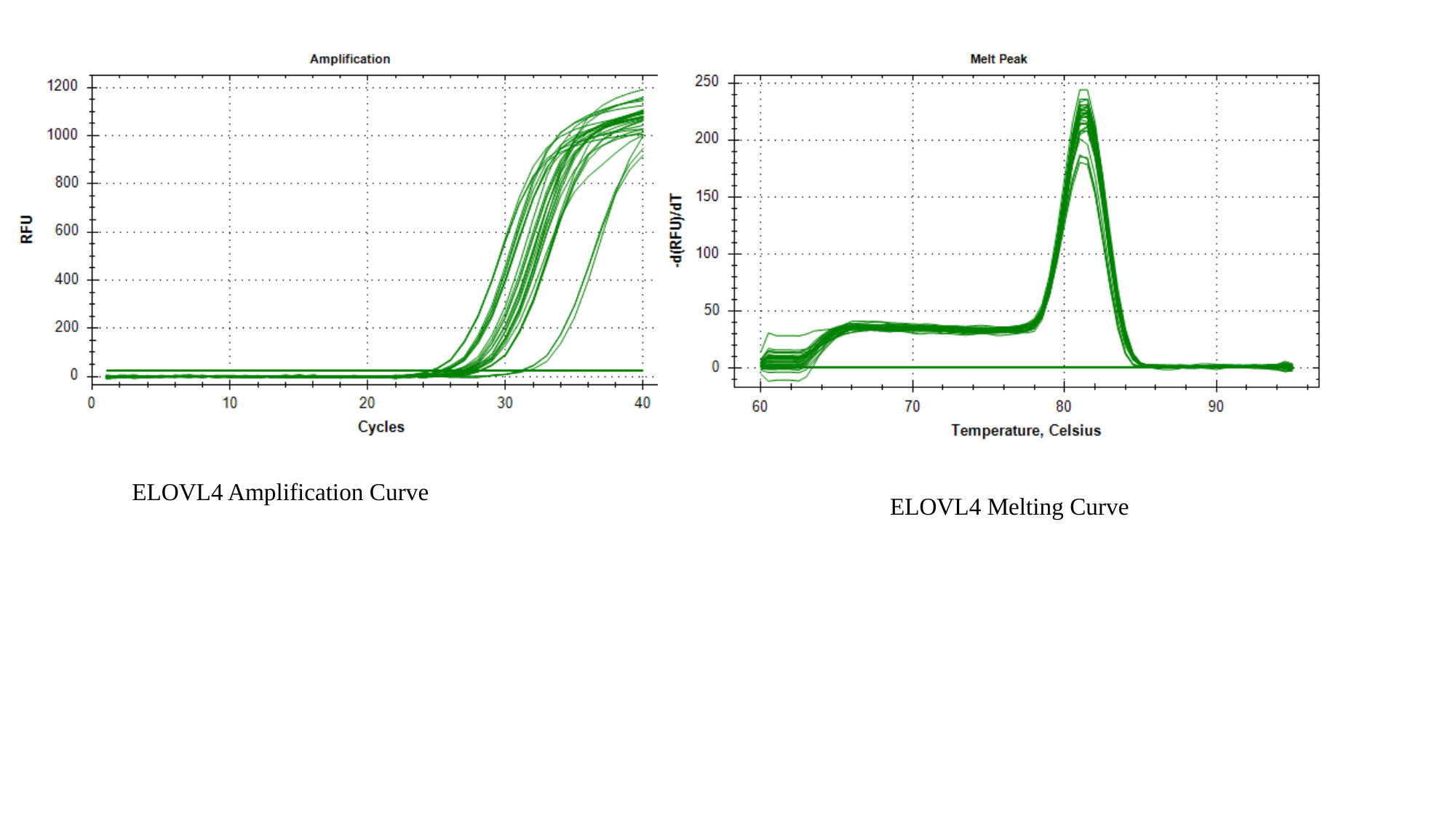

ELOVL4 Amplification Curve
ELOVL4 Melting Curve

## Slide 2
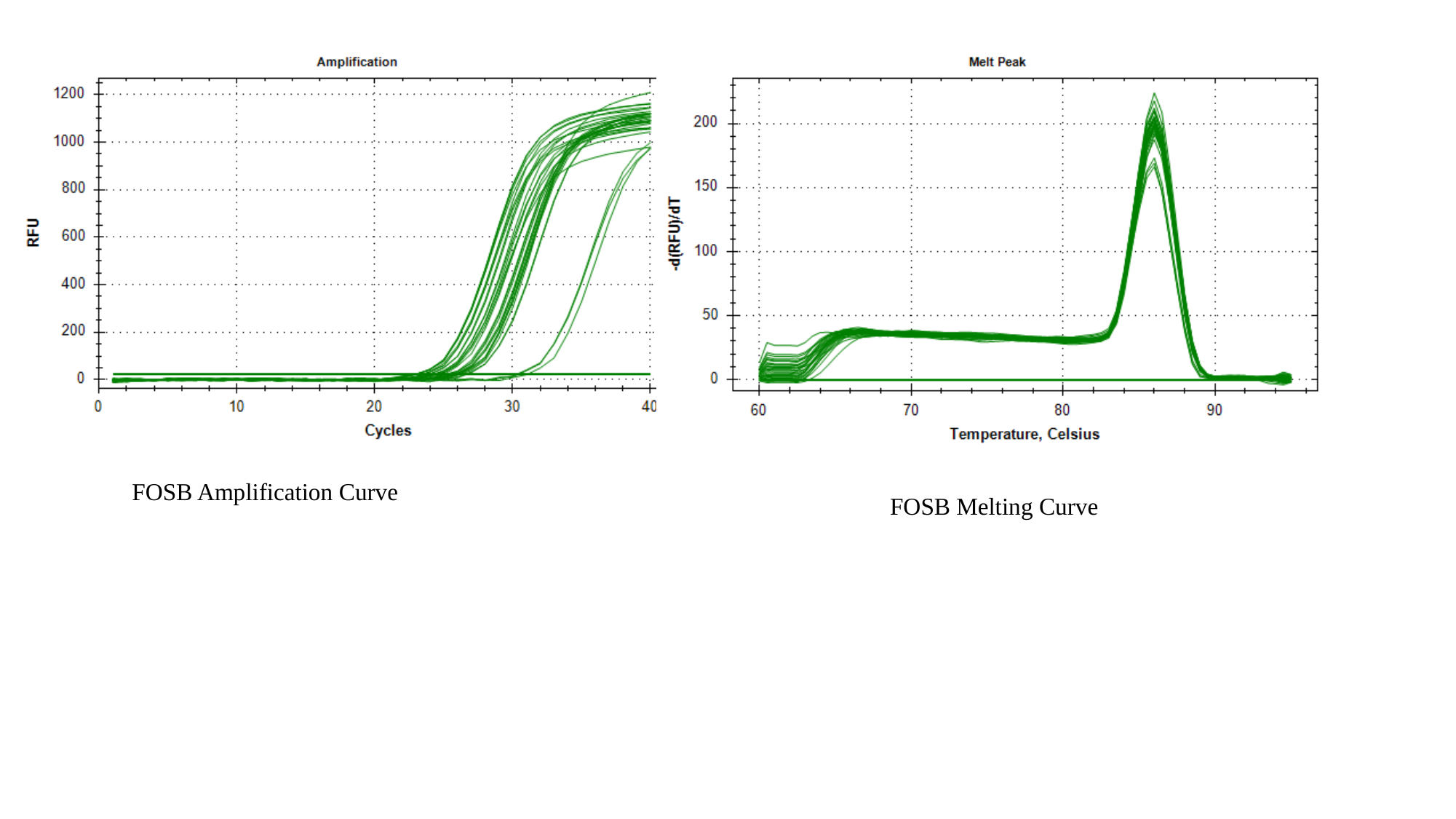

FOSB Amplification Curve
FOSB Melting Curve

## Slide 3
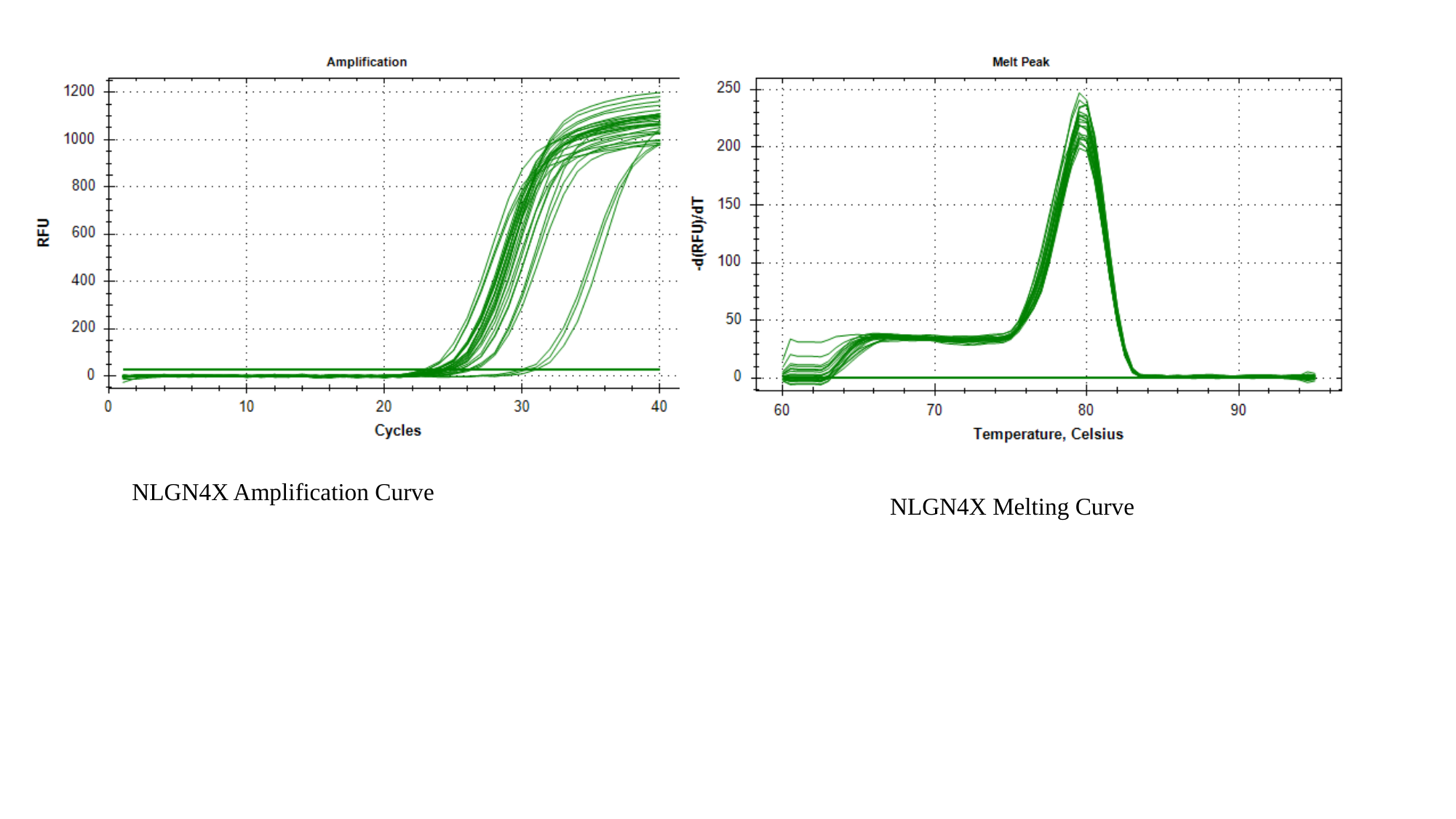

NLGN4X Amplification Curve
NLGN4X Melting Curve

## Slide 4
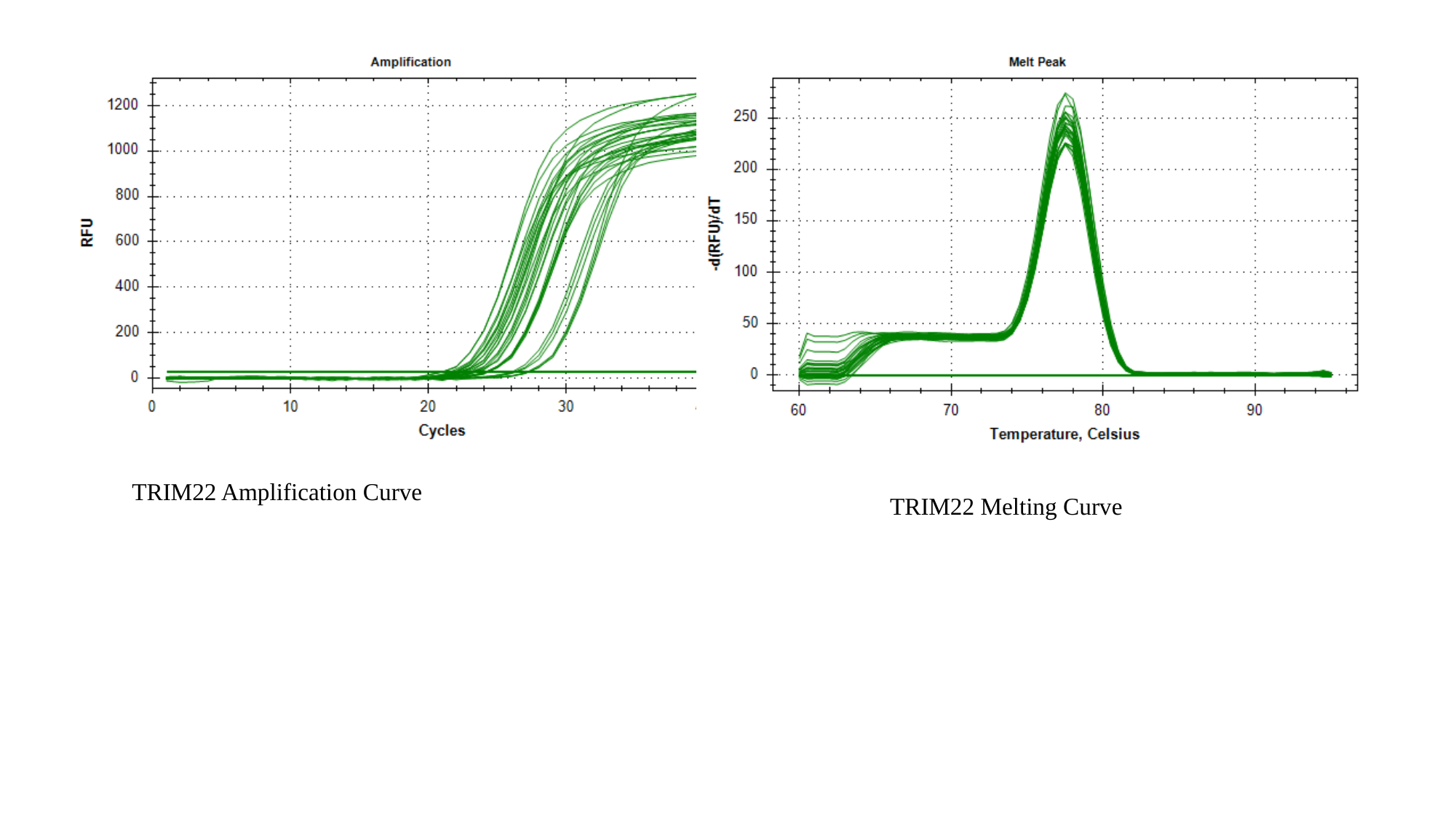

TRIM22 Amplification Curve
TRIM22 Melting Curve

## Slide 5
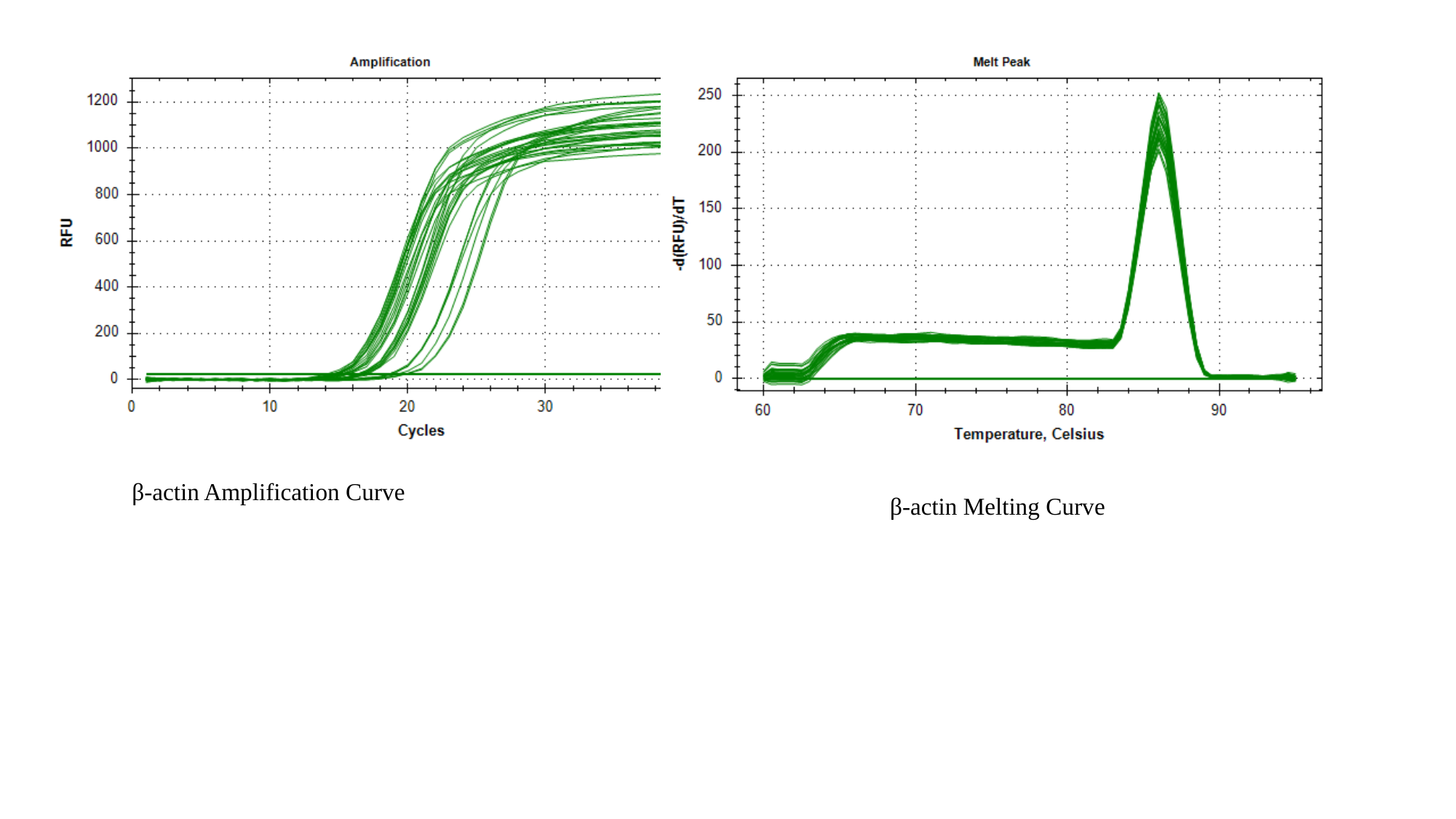

β-actin Amplification Curve
β-actin Melting Curve
